# Amount of Colicin Release in *Escherichia coli* Is Regulated by Lysis Gene Expression of the Colicin E2 Operon

**DOI:** 10.1371/journal.pone.0119124

**Published:** 2015-03-09

**Authors:** Andreas Mader, Benedikt von Bronk, Benedikt Ewald, Sara Kesel, Karin Schnetz, Erwin Frey, Madeleine Opitz

**Affiliations:** 1 Center for NanoScience, Faculty of Physics, Ludwig-Maximilians-Universität München, Geschwister-Scholl-Platz 1, Munich, Germany; 2 Institute for Genetics, Universität zu Köln, Köln, Germany; 3 Arnold-Sommerfeld-Center for Theoretical Physics and Center for NanoScience, Department of Physics, Ludwig-Maximilians Universität München, München, Germany; Arizona State University, UNITED STATES

## Abstract

The production of bacteriocins in response to worsening environmental conditions is one means of bacteria to outcompete other microorganisms. Colicins, one class of bacteriocins in *Escherichia coli*, are effective against closely related *Enterobacteriaceae*. Current research focuses on production, release and uptake of these toxins by bacteria. However, little is known about the quantitative aspects of these dynamic processes. Here, we quantitatively study expression dynamics of the Colicin E2 operon in *E*. *coli* on a single cell level using fluorescence time-lapse microscopy. DNA damage, triggering SOS response leads to the heterogeneous expression of this operon including the *cea* gene encoding the toxin, Colicin E2, and the *cel* gene coding for the induction of cell lysis and subsequent colicin release. Advancing previous whole population investigations, our time-lapse experiments reveal that at low exogenous stress levels all cells eventually respond after a given time (heterogeneous timing). This heterogeneous timing is lost at high stress levels, at which a synchronized stress response of all cells 60 min after induction via stress can be observed. We further demonstrate, that the amount of colicin released is dependent on *cel* (lysis) gene expression, independent of the applied exogenous stress level. A heterogeneous response in combination with heterogeneous timing can be biologically significant. It might enable a bacterial population to endure low stress levels, while at high stress levels an immediate and synchronized population wide response can give single surviving cells of the own species the chance to take over the bacterial community after the stress has ceased.

## Introduction

**Fig 1:**
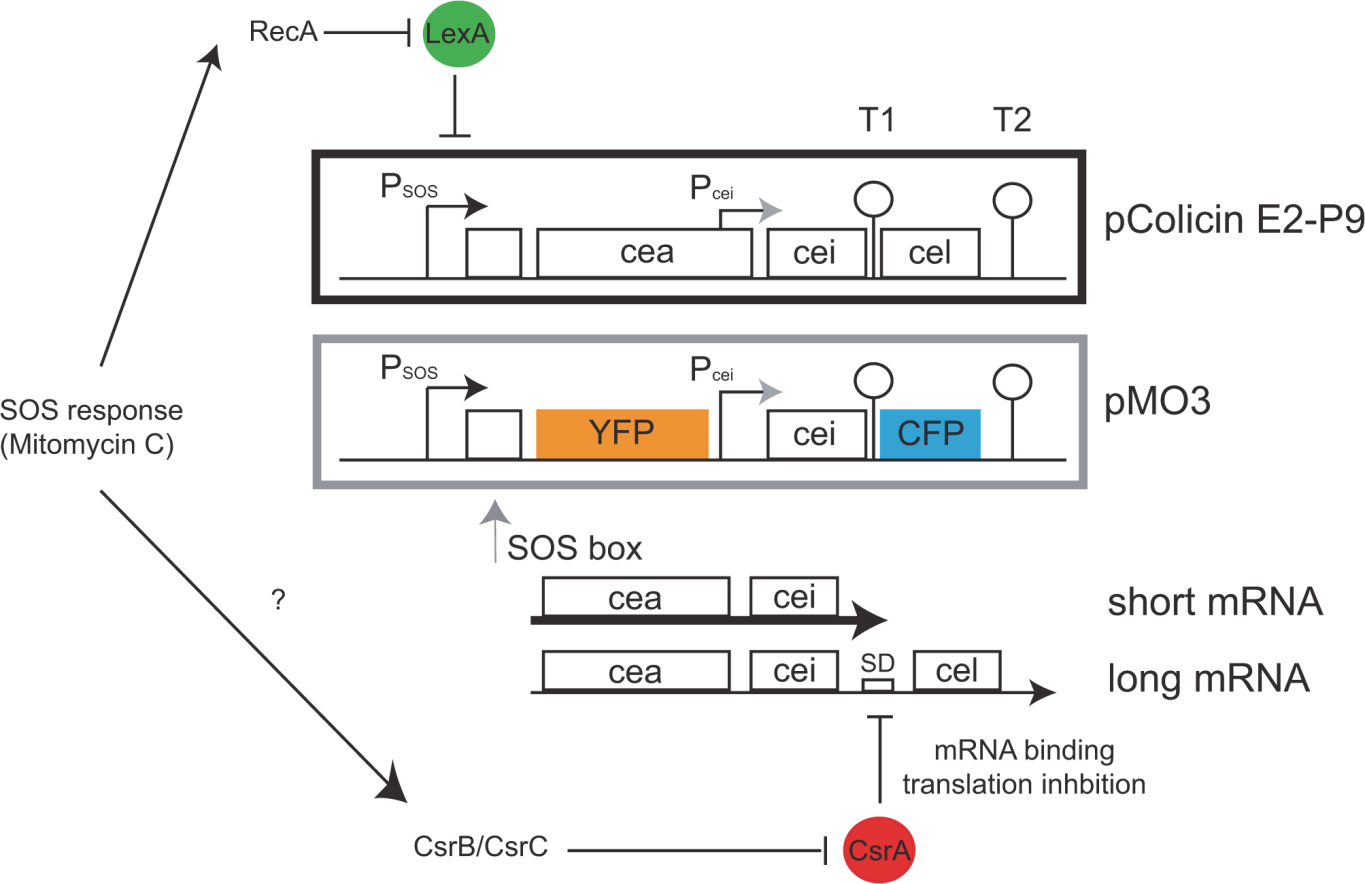
Gene regulation scheme for the original Colicin E2 system and pMO3. In the black box of the figure the original Colicin E2 operon of the plasmid pColE2-P9 as described in Pugsley *et al*. [9] is presented. It includes three genes: *cea* encoding the colicin activity protein, *cei* the immunity protein, and *cel* the lysis protein. The complete operon stands under the control of the SOS promoter (P_SOS_) and is only expressed after SOS induced cleavage of LexA from the SOS box located next to P_SOS_. A second promoter, the constitutive P_cei_ responsible for *cei* expression, is located prior the *cei* gene. If LexA is released from the SOS box, two different mRNAs are produced: a short mRNA encoding *cea* and *cei* is produced in abundance, and a long mRNA that is only produced if the transcriptional terminator T1 can be overcome. This long mRNA is then post-transcriptionally regulated by the mRNA binding protein CsrA. CsrA inhibits translation of the *cel* gene via binding to two CsrA binding sites present in the Shine Dalgarno sequence (SD) and the transcriptional terminator T1 upstream of the *cel* gene. The grey box depicts the same Colicin E2 operon with *cea* and *cel* exchanged by fluorescent proteins (FP) YFP and CFP, present on pMO3. Please note that while in strain EMO3-C both plasmids pColE2-P9 and pMO3 are present, strain EMO3 only carries pMO3.

Bacteria possess several mechanisms enabling them to respond to changing and unfavorable environmental conditions or to outcompete other bacteria [1–5]. One particular mechanism is the production and the release of toxins such as bacteriocins. Colicins are the best characterized group of bacteriocins [6–8] produced by *Escherichia coli* and active against closely related *E*. *coli* bacteria or other members of the *Enterobacteriaceae* [5]. Experimental studies focus on the mechanism of colicin release [9–11], colicin uptake by strains sensitive to the bacteriocin [12, 13], or the evolutional and ecological importance of colicins [4, 5, 14, 15]. In contrast, the majority of the theoretical investigations have been studying the interplay of colicin-producing bacteria with bacteria that are sensitive to or resistant against the bacteriocin [16–21]. Toxins, such as the bacteriocin Colicin E2 of this study, are plasmid encoded and expressed from operons under the control of an SOS promoter [8, 22, 23]. The Colicin E2 operon comprises three genes: *cea* (the colicin activity gene), *cei* (the immunity gene) and *cel* (the lysis gene) (Fig. 1). This operon is only expressed upon induction of the SOS response by e.g. DNA damage [8, 24]. When the Colicin E2 operon is expressed, two different mRNA transcripts can be found. The shorter transcript, which is transcribed at relatively high levels, includes the *cea* and *cei* gene. The longer transcript comprising all three genes, is rarely expressed only when the transcriptional terminator T1 can be overcome [8, 25]. For colicin E7 it was shown that translation of the *cel* gene is further regulated post-transcriptionally by the mRNA binding protein CsrA [25]. CsrA itself is further regulated by the sRNAs CsrB and CsrC. These sRNA bind the CsrA protein and can thereby reduce the amount of free CsrA if sRNA expression is high [26–28]. Since the operons of Colicin E2 and E7 show a high sequence homology [29, 30] and all regulatory elements present in the colicin E7 operon are also present in the Colicin E2 operon, it is assumed that CsrA is also inhibiting the translation of the lysis gene of the Colicin E2 operon. The co-expression of the colicin gene *cea* and the immunity gene *cei* is necessary, since the immunity protein ensures inactivity of the colicin as long as the colicin-immunity protein complex is present within the cell [8]. This colicin-immunity complex is highly stable [31] and can only be secreted when the lysis gene is expressed [9–11], which leads to the subsequent death of the bacterial cell, secreting the colicin [9]. Under exponential growth conditions colicins are not or only rarely [32] synthesized since the colicin operon is repressed by the protein LexA (transcriptional control [8]). Upon DNA damage, that can be induced by UV irradiation or the addition of SOS-agents such as the chemical Mitomycin C [24, 33–35], the RecA protein binds to ssDNA and forms active RecA nucleo-proteinfilaments [36]. In this active state, RecA promotes auto-cleavage of LexA dimers [36]. Subsequently, the amount of free LexA dimers is no longer sufficient to repress the Colicin operon and the toxin is expressed at high rates [37]. In consequence, a large fraction of cells is expressing the colicin. It was shown that genes regulated by the *E*. *coli* SOS repressor LexA exhibit a heterogeneous expression [38, 39] upon nutrient starvation that is primarily established by stochastic factors [38].

While whole population studies revealed the heterogeneous expression of several colicin operons [24, 38, 39], still a detailed and quantitative analysis about the expression dynamics on the single cell level is lacking. Knowledge of the heterogeneous expression dynamics is crucial to gather information about the biological significance of the observed phenotypic heterogeneity. To address this question, we performed quantitative time-lapse fluorescence microscopy under natural and stress-induced conditions. Using fluorescent reporter constructs, we thereby discriminate between the expression of the colicin gene (*cea*) and the lysis gene (*cel*) necessary for colicin release (Fig. 1), as we expect to observe differences in the heterogeneous expression of these genes due to the additional post-transcriptional regulation of the *cel* gene. Interestingly, we find both genes to be co-expressed in strain EMO3-C. While at low stress levels all cells eventually respond with time, a phenomenon known as heterogeneous timing, this heterogeneous timing is lost at high stress levels in favor of a synchronized response of the whole population. Furthermore, using a mutant strain unable to produce and release the colicin, we demonstrate that the amount of colicin released into the surrounding environment is dependent on *cel* gene expression and not due to a specific colicin concentration threshold.

## Materials and Methods

### Strains and plasmids used in this study

The strains and plasmids used in this study are listed in Table 1. To analyze expression of the genes *cea* (colicin activity protein) and *cel* (lysis protein) of the Colicin E2 operon, we created a mutant strain carrying the additional plasmid pMO3. This plasmid, pMO3, represents a double reporter plasmid. It carries the complete Colicin E2 operon, but the genes *cea* and *cel* have been replaced by genes coding for the fluorescence proteins (FP) mVenus (YFP) and mCerulean (CFP), respectively (Fig. 1). Plasmid pMO3 was created as described in the following: We used the synthetically created vector pEX-K-colicin (MWG Eurofins, Ebersberg) that harbors the complete sequence of the Colicin E2 operon (NCBI M29885) with the exception of the *cea* gene. In addition, slight sequence changes have been introduced into the Colicin E2 operon present in pEX-K-colicin to enable further cloning with appropriate restriction sites. These changes were designed without altering the regulatory elements present in the original Colicin E2 operon.

**Table 1:** Strains and plasmids used in this study.

**Name**	**Description**	**Referenz**
BZB 1011 E2C	pColE2-P9	[17]
BZB 1011	Same as BZB 1011 E2C without the colicin plasmid	[17]
EMO3-C	BZB 1011 E2C pMO3	This study
EMO3-S	BZB 1011 pMO3	This study
pEX-K-Colicin	Commercially obtained vector pEX-K-Colicin (MWG Eurofins, Ebersberg) harboring the complete Colicin E2 operon with the exception of cea. Slight sequence changes have been incorporated to allow introduction of necessary restriction sites.	This study
pColE2-P9	Native colicin plasmid with complete Colicin E2 operon comprising the *cea*, *cei* and *cel* gene	[9]
pKES258	mVenus	unpublished
pKEHB1	mCerulean (A206K)	unpublished
pMO1	Ligation of ClaI—HindIII fragment of pEX-K-Colicin with the backbone of pBAD24GFP	This study
pMO2	Ligation of pMO1 with a mVenus PCR fragment (restriction sites EcoRI and SacI) of pKES258	This study
pMO3	Exchange of *cel* gene in pMO2 with a CFP PCR fragment (restriction sites BamHI and AgeI) of pKEHB1	This study

A *ClaI—HindIII* fragment of pEX-K-Colicin (Table 1) was ligated with the backbone of pBAD24-GFP [40], to combine the modified Colicin E2 operon of pEX-K-Colicin with the backbone of the multi copy pBAD24-GFP plasmid. The plasmid pBAD24-GFP has an average copy number of 55 plasmids per cell [40] and a cell-to-cell variation of about 7.5 plasmids [40], decreasing cell-to-cell variations in fluorescence intensity that might be due to variations in plasmid copy number to a minimum. The resulting vector pMO1 includes now all regulatory elements of the original Colicin E2 operon as well as the Ampicillin resistance and the *ori* of pBAD24-GFP. In order to introduce the fluorescence protein mVenus at the site of the *cea* gene into pMO1, we obtained this FP via PCR from pKES258 (unpublished, Table 1) using the primer P1 and P2 (Table 2). This PCR fragment of pKES258, including the FP mVenus and appropriate restriction sites *EcoRI* and *SacI* for integration in pMO1, was generated and subsequently integrated in pMO1. The resulting new vector pMO2 expresses instead of the original bacteriocin Cea, mVenus, a YFP derivate fluorescent protein and can therefore be used as a reporter construct for the expression of *cea*-mRNA. To achieve a double reporter plasmid that acts as a reporter for toxin as well as for lysis gene mRNA production, the *cel* gene of pMO2 was exchanged with a *AgeI—BamHI* PCR product (primer P3 and P4, template pKEHB1 unpublished, Table 1 and Table 2) containing mCerulean, a CFP fluorescent protein. This new plasmid was named pMO3 (Fig. 1) and represents the double reporter for *cea* (monitored via YFP) and *cel* (monitored via CFP) expression. Correct construction of all plasmids was confirmed by sequencing. The plasmid pMO3 was than transformed into strain BZB 1011 E2C (carrying the original colicin producing plasmid pColE2-P9, with a copy number of about 10–20 [8, 41]) and strain BZB 1011 lacking pColE2-P9 [17]. The resulting strains were named EMO3-C (colicin producing) and EMO3-S (not able to produce the colicin) respectively (Table 1).

**Table 2:** Primers used in this study.

**Name**	**Sequence**	**Purpose**
P1	5´ atgcGAATTCatgagcaagggcga 3´	mVenus [fwd]
P2	5´ tagcGAGCTCttacttgtacagctcg 3´	mVenus [rev]
P3	5´ attaACCGGTatggtcagcaa gggcg 3´	mCerulean [fwd]
P4	5´ atgcGGATCCttacttgtacagctcg 3´	mCerulean [rev]

### Growth conditions

EMO3-C and EMO3-S were grown overnight at 37°C in M63 minimal medium supplemented with 0.5% Glycerol as a carbon source, and with 100μg/ml Ampicillin (Carl Roth, Germany) when needed. Overnight cultures were diluted to an OD_600_ of 0.05. The subsequent day-culture was allowed to grow until it reached an OD_600_ of 0.2, which represents the beginning of the exponential growth phase. Bacteria were then induced with different concentrations of the SOS agent Mitomycin C (Carl Roth, Germany) and used for the further investigations as indicated in the main text.

### Single cell time-lapse microscopy and image analysis

For single cell time-lapse microscopy Ibidi μ-slides VI^0.4^ (Ibidi GmbH, München) were coated with 50μl Poly-L-Lysine (BIOCHROM, Berlin) for at least two hours. After that, the slides were rinsed three times with water and two times with M63 minimal media. Bacteria were grown and induced with Mitomycin C (MitC) as described above and diluted again, if necessary, prior transfer to the microscopic slide to obtain optimal cell densities for single cell analysis. 50μl of the diluted culture were allowed to attach to the surface of the slide for 7.5 minutes at 37°C. Then the channel was rinsed with M63 minimal media for four times to remove bacteria that had not attached to the surface. The microscopic slide was then transferred to an inverse microscope, Axiovert 200M (Carl Zeiss, Germany) equipped with an Andor camera and a Zeiss EC Plan-Neofluar 100x/1.3 Oil immersion objective. For YFP detection a filterset with a beamsplitter BS520, an excitation bandpass HC500/24 and an emission bandpass HC 542/27 was used. The filterset for CFP detection was a HC filterset with an emission filter 483/32, a beamsplitter BS458 as well as an excitation filter 438/24. With mutant strains carrying either the YFP or CFP reporter, exclusively, we confirmed that no cross-talk between the YFP and CFP channel was present using the filter sets described above. To minimize fluorescence variations deriving from day to day deviations of the fluorescence lamp, the stability of the absolute fluorescence values were verified daily using a microscope image intensity calibration kit (Invitrogen, FokalCheck fluorescence microscope test slide #3). The obtained data sets were corrected accordingly. The micromanager, an open source program (version 1.3), was used for image acquisition [42]. After the first image, the channel was flushed with media equipped MitC in the appropriate concentration. Subsequently, an image was taken every 15 min. Images were analyzed using the plugin Cell evaluator [43] of the freeware ImageJ. Only live cells that were fully lying within the bright field image and were not in the process of cell division were considered. For each time-point or MitC concentration at least 85 bacteria were analyzed. All of our experiments have been performed in duplicates or triplicates on at least two different days. General data analysis was performed using IgorPRO 6.22 and Adobe CS5 Software.

### Definition of Threshold

To quantify the amount of cells expressing the fluorescent proteins YFP or CFP (representing the expression of the genes *cea* and *cel* of the Colicin E2 operon), respectively, we needed to set a threshold to distinguish cells expressing a fluorescent protein at high levels from those not or only weakly expressing the fluorescent protein. We defined the threshold as denoted in the following. For the mean fluorescence intensity (FI) value of the first three experimental time-points we observe that no cell starts to express either YFP or CFP. Therefore, we take this mean FI value as the basal fluorescence of the individual cell (S1 Fig.). Cells were defined as switching into the ‘ON’ state, if their individual FI level is five times higher than their basal fluorescence. These cells can then be clearly distinguished from cells in the ‘OFF’ state. The time-point *t* of switching into the ‘ON’ state is defined as the time-point when cells overcome this switching threshold (S1 Fig.).

## Results

### Similar expression dynamics of *cea* and *cel* gene expression in strain EMO3-C

Several groups have studied the heterogeneous expression of colicin operons [38, 44], focusing on population wide studies that reveal the average response of the whole population at a given time-point [24, 32, 34, 45–47]. Here, we wanted to go a step further and analyze the expression dynamics of one of these colicin operons, namely the Colicin E2 operon, using quantitative fluorescence time-lapse microscopy. Furthermore, we wanted to investigate whether there are differences in the heterogeneous expression of the *cea* (colicin) and *cel* (lysis) gene of this operon. *Cel* gene expression was shown to be post-transcriptionally regulated by CsrA in the colicin E7 operon [25], which is not the case for *cea* gene expression. Similar to the colicin E7 operon, the Colicin E2 operon possesses CsrA binding sites in the *cel* gene Shine-Dalgarno sequence (Fig. 1). Binding of CsrA to these sites could lead to *cel* translation inhibition. We therefore expected to find differences in the expression dynamics and the heterogeneous expression of the *cea* and *cel* gene.

To study heterogeneous expression of the *cea* and *cel* gene we introduced the double reporter plasmid pMO3 in addition to the original plasmid pColE2-P9 into *E*. *coli* strain BZB 1011 E2C [17]. The resulting strain was named EMO3-C (Table 1). This double reporter plasmid carries the complete Colicin E2 operon, but the genes *cea* and *cel* have been replaced by genes encoding the fluorescence reporters YFP and CFP, respectively (Fig. 1, Materials and Methods). In this double reporter plasmid, all regulatory elements for transcriptional, as well as for post-transcriptional regulation are present. This includes the SOS box for LexA binding, the transcriptional terminators T1 and T2, as well as the ribosome-binding site (RBS) of *cel* and CsrA binding sites. Thus any regulatory mechanism, such as a probable colicin regulatory feedback [15, 41], should not be affected in the double reporter plasmid, and the dynamics of fluorescent reporter expression may be assumed to directly reflect the dynamics of *cea* and *cel* gene expression.

**Fig 2:**
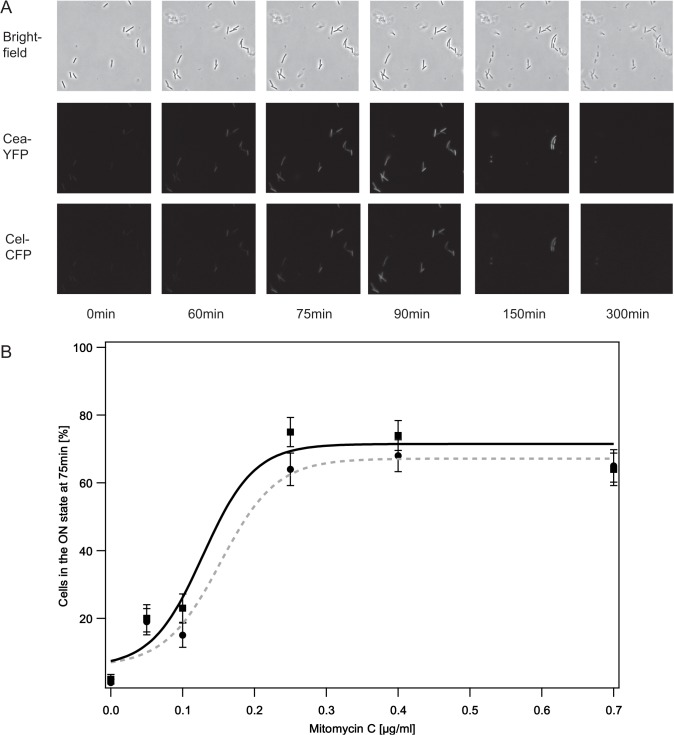
Percentage of cells in the ON state at a distinct time-point. A: Example time series of brightfield, YFP (*cea* expression) and CFP (*cel* expression) fluorescence images acquired at the denoted time-points at a MitC concentration of 0.4 μg/ml. B: Percentage of cells in the ON state for *cea* expression (black squares) as well as for *cel* expression (black circles) 75 min after induction with the SOS agent MitC. The data were fitted with a sigmoid function (solid black line *cea* expression, dashed grey line *cel* expression, S3 Table), the errorbars denote the standard error.

Using fluorescence time-lapse microscopy, we studied the parallel expression of YFP and CFP (representing *cea* and *cel*, respectively) in individual bacterial cells. Cells were taken in the early exponential phase and subsequently analyzed either under natural conditions or in the presence of the SOS agent Mitomycin C (MitC) at various concentrations (Material and Methods). To ensure that any differences in gene expression of the YFP and CFP reporter are not due to differences in their maturation times, we measured the maturation times of these fluorescence reporters in our strain EMO3-C (S1 Text), using a standard protocol [40, 48]. Both fluorescence proteins matured similar fast with a maturation time of less than 12 min, which is comparable to literature values [49, 50] and below the experimental resolution time of 15 min. Despite our expectations, we found the expression dynamics of the *cea* and *cel* gene to be very similar, if not identical in strain EMO3-C (Fig. 2 A). This co-expression of the *cea* and *cel* gene could be observed for all applied MitC concentrations as well as for the un-induced (natural) case, indicating that a post-transcriptional regulation that is only affecting *cel* gene expression did not occur or is not detectable under the experimental conditions used in this study. To compare our time-lapse microscopy data with previous whole population studies in a quantitative way, we introduced a threshold YFP or CFP fluorescence value (*cea* or *cel* concentration) that separated non-expressing cells with only low fluorescence intensities (FI) from highly expressing cells with high fluorescence values. These highly expressing cells were then considered to be in the ‘ON’ state (Materials and Methods, S1 Fig.). The percentage of cells in the ‘ON’ state (*cea* and *cel* gene expression) for various concentrations of the SOS agent MitC at 75min after induction with MitC is shown in Fig. 2B. This time-point was chosen, as whole population studies performed with strain EMO3-C revealed (data not shown) that for high MitC concentrations the maximum response could be observed at 75min after induction with this SOS agent. This observation was in accordance with literature values that stated that 60 to 90 min after stress induction the maximum average colicin expression could be determined [8, 33, 47]. As seen in earlier studies [24, 33, 47], we found that the amount of cells in the ‘ON’ state increases with the exogenous stress level, saturating at 75% and 68% for *cea* and *cel* gene expression, respectively (S1 Table and S2 Table). An increase of MitC concentrations exceeding 0.25 μg/ml does thereby not lead to a further increase in the fraction of cells in the ‘ON’ state. Multidrug efflux pumps such as tolC [51] could thereby affect the MitC concentration at which this saturation effect can be observed, as they reduce the intracellular MitC concentration [51, 52]. Again, we were not able to observe a significant difference in the fraction of cells in the ‘ON’ state for *cea* and *cel* gene expression in strain EMO3-C. In the following, we therefore present *cea* (YFP) gene expression data in the main manuscript. The equivalent data for *cel* (CFP) gene expression can be found in the supporting information.

### Colicin E2 expression is characterized by heterogeneous timing at low exogenous stress levels, a phenomenon that is lost at high exogenous stress levels

**Fig 3:**
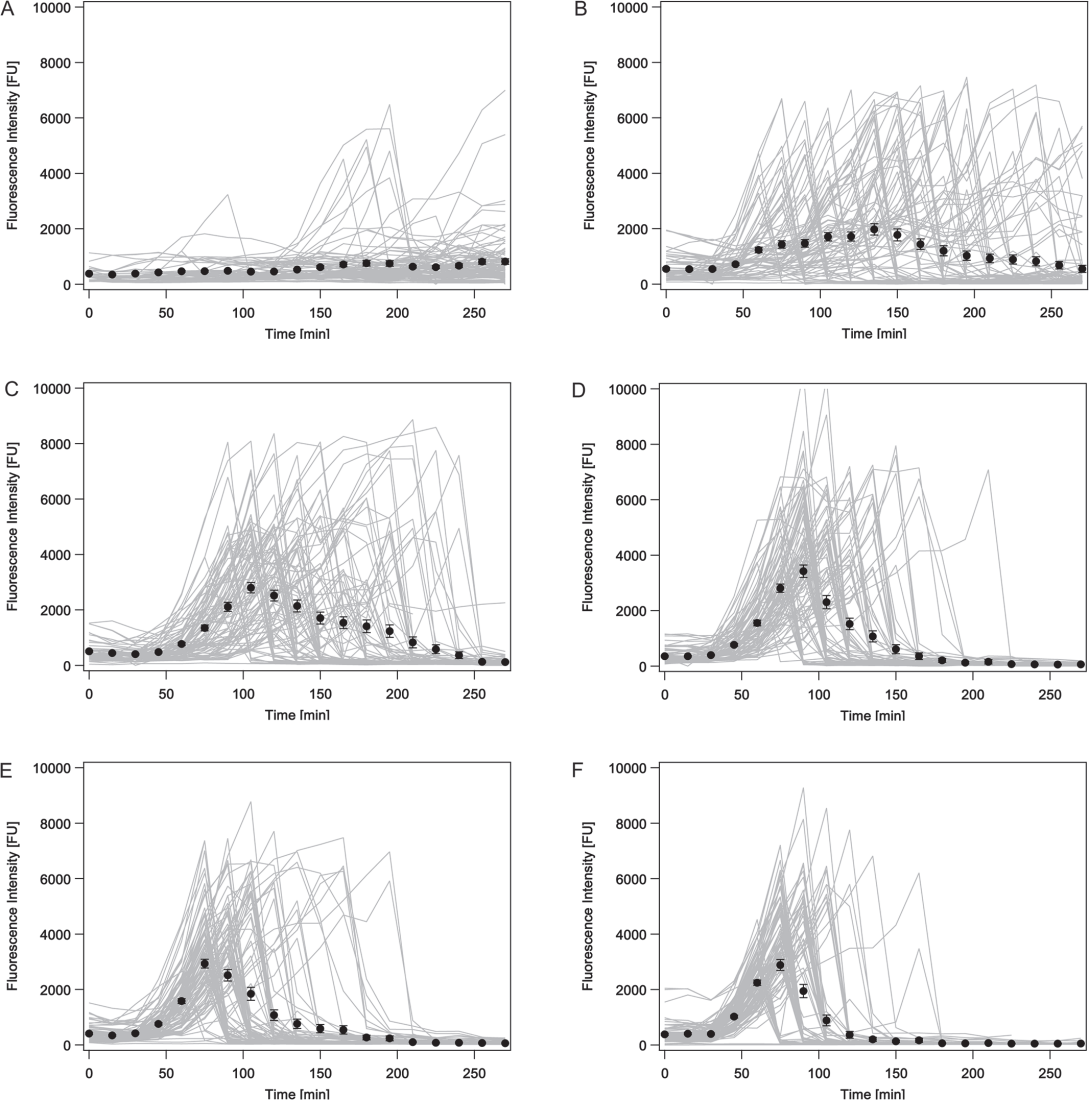
Single cell time traces of *cea* expressing cells. A—F Fluorescence intensity (FI) over time for single cells expressing YFP (depicts *cea* expression, grey lines) as well as the mean fluorescence intensity (black circles) of all cells at each single time-point is shown, the errorbars denote the standard error of the mean. A: no MitC added, B: 0.05 μg/ml MitC, C: 0.1 μg/ml MitC, D: 0.25μg/ml MitC, E: 0.4 μg/ml MitC, F: 0.7 μg/ml MitC. The corresponding data for *cel* gene expression are given in S2 Fig.

**Fig 4:**
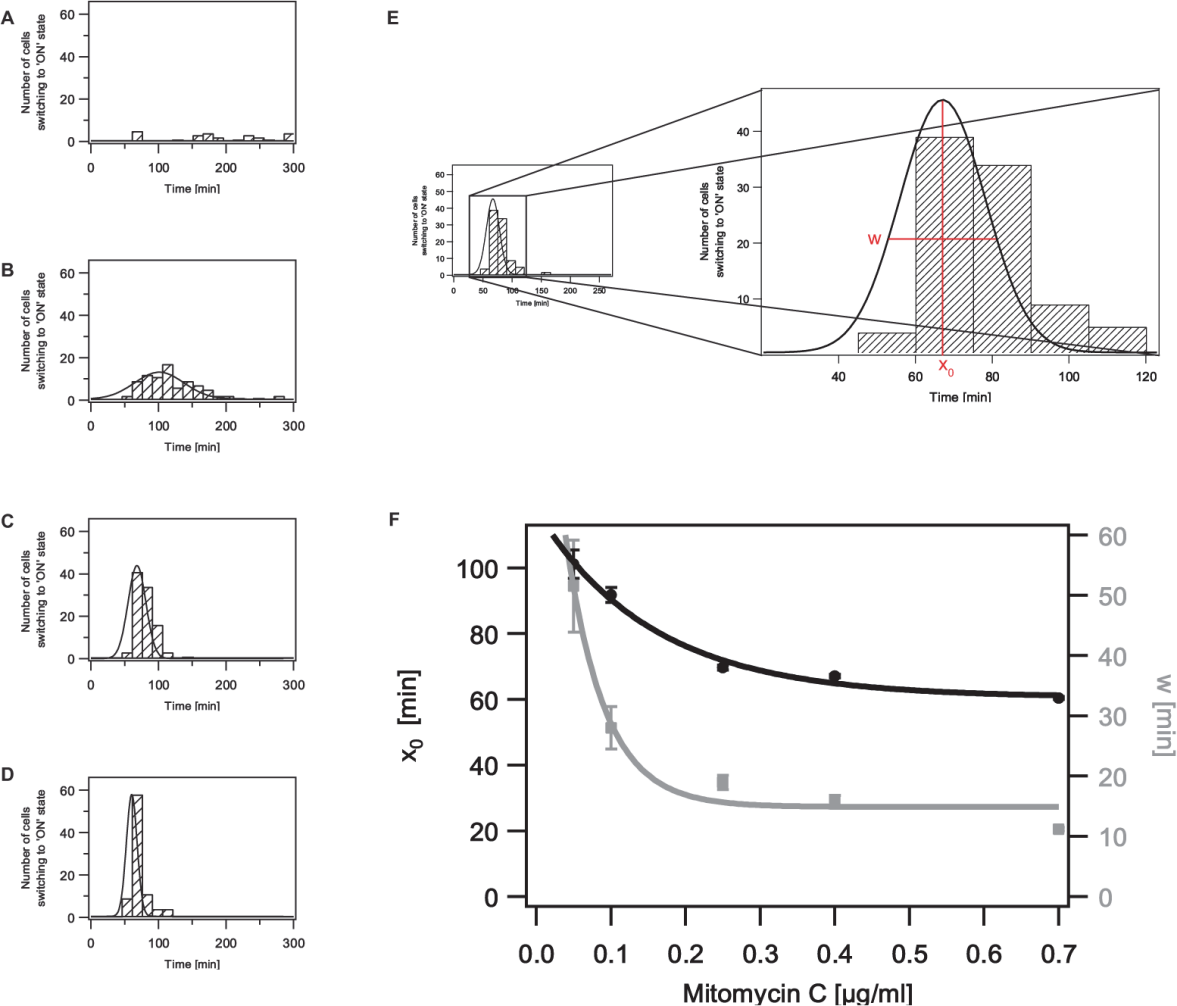
Determination of the switching window of *cea* expressing cells in dependence of different MitC concentrations. A—D: Histograms of the number of cells switching into the ‘ON’ state at every time-point. The histograms are given for different MitC concentrations (A: 0.0 μg/ml, B: 0.05 μg/ml, C: 0.25 μg/ml, D: 0.7 μg/ml) The black lines denote Gaussian fits to these histograms (S4 Table). Please note, due to the small number of cells in the ‘ON’ state a Gaussian fit could not be applied for 0.0 μg/ml MitC. E: Close up of a histogram of the number of cells switching to the ‘ON’ state at every time-point. Two parameters are of interest: w, the FWHM of the applied Gaussian fit representing the time-period in which switching into the ‘ON’ state occurs and x_0_ the maximum of the fit, representing the time-point at which the maximal number of cells responds to the stressor MitC. F: Time-point of maximal switching into the ‘ON’ state (x_0,_ black circles) of *cea* expression for different MitC concentrations, fitted by an exponential decay (black line, S5 Table). Switching window of *cea* expression (w, grey squares) at different MitC concentrations, fitted by an exponential function (grey line, S5 Table) The errorbars show the errors of the fits of the corresponding histograms. The corresponding data for *cel* gene expression are given in S3 Fig.

As seen in whole population studies [24, 32, 33, 47], our single cell time-lapse microscopy revealed that at a given time-point only a fraction of the cells actually responds to the applied stressor MitC. The question remained elusive whether with time eventually all cells respond and whether or not this takes place in a homogenous fashion with all cells responding with the same intensity. In contrast to previous whole population studies, we could address this question by the performance of single cell time-lapse microscopy. For uninduced (natural) conditions, the analysis of *cea* (YFP) expression over time in single cells revealed, that in the exponential growth phase only a few cells express *cea* (YFP), followed by a significantly higher fraction of cells expressing *cea* with entry into the stationary growth phase (Fig. 3 A). With increasing MitC concentration, more and more cells express *cea* (YFP) and do so at earlier time-points (Fig. 3 B-F, equivalent data for *cel* gene expression can be found in S2 Fig.). Interestingly, already at the very low MitC concentration of 0.05μg/ml, within the time-course of five hours, nearly all cells start expressing *cea*, a phenomenon described as heterogeneous timing. While for low MitC concentrations (0.05 and 0.1 μg/ml, Fig. 3B, C) cells start expressing *cea* over a broad time-period, at high MitC concentrations *cea* expression occurs in a much shorter time-window. We investigated this quantitatively and analyzed the number of cells switching into the ‘ON’ state with time (Fig. 4 A-D) (for threshold definition, see Materials and Methods, the equivalent data for *cel* gene expression are given in S3 Fig.). We find that both the time-point of maximal switching as well as the time-period of switching (Fig. 4E) decrease exponentially with increasing MitC concentration (Fig. 4F). At the lowest investigated MitC concentration cells respond over a time-period of more than 200 min. The maximal response can be observed at 101 ± 4 min. At 0.7 μg/ml MitC all cells respond within a time-window of less than 15 min and the maximal response can already be observed at 60 ± 0.5 min (S3 Table). In summary, our data reveal that the heterogeneous timing observed at low MitC concentrations is lost at high exogenous stress levels, where a synchronized stress response can be observed.

### The amount of colicin release is regulated by *cel* gene expression

**Fig 5:**
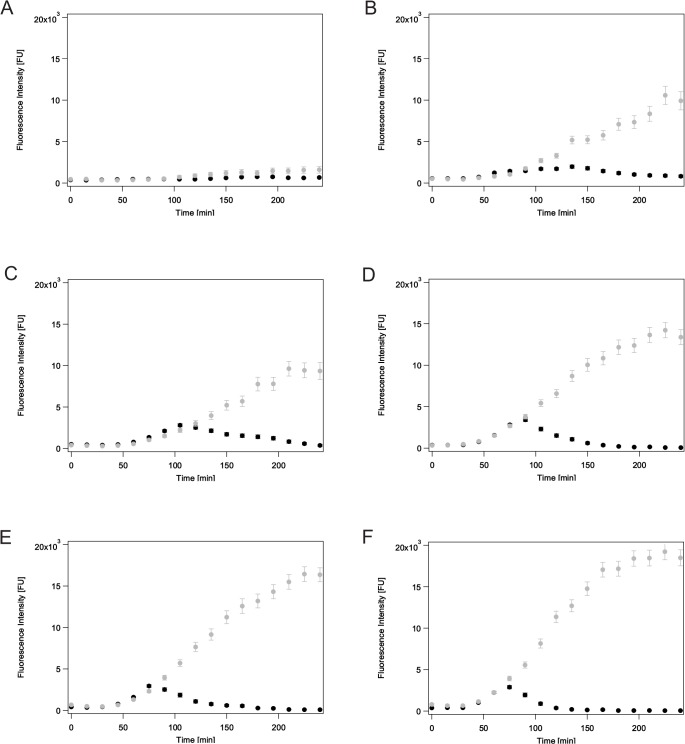
Comparison of YFP (*cea*) expression in EMO3-C and EMO3-S. A-F Mean fluorescence intensities for YFP (*cea*) expression over time for different MitC concentrations (A: 0.0 μg/ml, B: 0.05 μg/ml, C: 0.1 μg/ml, D: 0.25 μg/ml, E: 0.4 μg/ml and F: 0.7 μg/ml). EMO3-C harboring two plasmids (pMO3 and colicinE2-P9) is depicted in black circles; EMO3-S (with pMO3 only) is displayed in grey squares. The errorbars denote the error of the mean. The corresponding data for *cel* gene expression are given in S4 Fig.

Without MitC induction we observed a mean FI (*cea* expression) of cells in the ‘ON’ state of about 2905 FU (S1 Table). This value significantly increased upon MitC induction and was comparable for all MitC concentrations with values ranging from 4592 to 5138 FU (S1 Table), indicating that the same amount of colicin is produced independently of the inducing MitC concentration. The question remained how the achievement of this maximal colicin amount is regulated. Is a specific *cea* threshold present that induces cell lysis and with it colicin release? To address this question, we transferred the pMO3 plasmid into a second strain lacking the original pColE2-P9 plasmid. This strain, named EMO3-S (Materials and methods, Table 1), was therefore not able to produce the colicin and the lysis protein. We found that *cea* expression was similar for EMO3-C and EMO3-S up to a specific time-point. This time-point coincided with the time-point at which the maximal average fluorescence intensity could be observed (Fig. 3, black dots), as well as the time-point of maximal switching of strain EMO3-C (Fig. 4, S4 Table). While in the EMO3-C strain the mean YFP fluorescence decreased down to zero after this time-point, due to cell lysis, in the EMO3-S strain that is not able to lyse, a further increase of the mean YFP fluorescence was observed (Fig. 5, the equivalent data for *cel* gene expression are given in S4 Fig.). This finding was independent of the applied MitC concentration. These data clearly show that the amount of colicin released into the surrounding environment is not dependent on a particular colicin concentration, but is regulated by *cel* gene expression leading to cell lysis.

## Discussion

In this work, we present the first quantitative analysis of the expression dynamics of the Colicin E2 operon in *E*. *coli*. Using single cell time-lapse microscopy, we thereby distinguished between the expression dynamics of the *cea* gene encoding the toxin Colicin E2 and the *cel* gene responsible for colicin release. As described in the introduction, two different types of mRNA can be produced: long and short mRNA, but only the long mRNA also includes the *cel* gene. In addition, binding sites for the mRNA binding protein CsrA are present in the RBS of the *cel* gene, introducing post-transcriptional regulation of *cel* gene expression via translation inhibition by CsrA. By exchanging the *cea* and *cel* genes with fluorescent reporter genes while keeping all regulatory elements, we were addressing two questions: a) is the heterogeneous gene expression of *cea* and *cel* different and b) do we see differences in the onset of *cea* and *cel* gene expression. In contrast to our expectations, we did not observe a significant difference in *cea* and *cel* gene expression in strain EMO3-C. In addition, the onset of gene expression was similar for both genes, indicating that post-transcriptional translation inhibition of the *cel* gene by CsrA did not occur or was not detectable under the experimental conditions used in this study. Although, CsrA has been described to be a high abundance protein [27, 53], the additional introduction of our double reporter plasmid could lead to a titration of CsrA and thereby affect inhibition of *cel* gene expression via CsrA in strain EMO3-C. Furthermore, two sRNAs, CsrB and CsrC have been reported to bind CsrA [26]. Increased expression of these sRNAs could reduce the amount of free CsrA, which in turn could affect the time-point of colicin release. Nevertheless, in agreement with previous whole population studies [24, 32, 47], our single cell time-lapse microscopy data confirm that the *cea* and *cel* genes of the Colicin E2 operon are heterogeneously expressed in the stationary phase. Similarly, whole population studies [38, 39, 44] of other colicins such as Colicin K revealed that these colicins are also heterogeneously expressed, indicating a common mechanism. With induction of the Colicin E2 operon by the SOS chemical MitC, the fraction of cells expressing either *cea* or *cel* increased with the applied MitC concentration and the cells’ response times decreased exponentially in dependence of the MitC concentration, saturating at 60 min. These data suggested that even very low exogenous stress levels can be sensed by individual cells, but cells are not able to produce and release the colicin prior to 60 min after induction by MitC.

Interestingly, our single cell time-lapse microscopy data revealed that even at the lowest tested MitC concentration nearly all cells started to express *cea* and *cel*, but did so within a time-period of about five hours. This could be seen as a new strategy of cells dealing with externally applied stress. At low stress levels only few cells produce the toxin and lyse in the beginning, while the remaining cells still have a chance to survive if the external stressor ceases. If at these low stress levels the stress continues, then eventually all cells will produce the colicin and undergo cell lysis. This behavior that eventually all cells respond with time, was previously observed for the utilization of the sugar arabinose in *E*. *coli* cells and described as heterogeneous timing [40]. Our data demonstrate that heterogeneous timing is characteristic for Colicin E2 expression at low exogenous stress levels.

Further increase of the MitC concentration and with it the applied stress level, lead to a loss of the observed heterogeneous timing. Firstly, all cells responded to the external stressor and heterogeneity (with one fraction of the population in the ‘ON’ state, while the other is remaining in the ‘OFF’ state) was completely lost. Secondly, all cells responded within a time-window of only 15min at high stress levels, leading to a synchronization of toxin production and release within the whole population. Thus our data reveal a transition from one response type to another at intermediate MitC concentrations. At low MitC concentrations, stochastic cell-to-cell variations of proteins of the SOS response [54], DNA repair mechanisms [55] or multidrug efflux pumps [51, 52] could cause the observed heterogeneous timing. These variations could be lost at high MitC concentrations that maximally induce the SOS response [56]. This transition between heterogeneous timing in favor of synchronization of colicin expression and release makes sense in the natural context: at high stress levels all cells respond and lyse. Thereby, if only one healthy cell survives, the chances of this cell to regrow a new population increase, as now closely related bacteria are ‘murdered’ due to the extremely high toxin concentration secreted and enough nutrients being left to ensure the creation of a new and healthy bacterial population.

The toxin concentration that is finally released into the surrounding environment does not seem to be dependent on a particular colicin threshold concentration, but rather regulated by the time-point of cell lysis due to *cel* gene expression. Although, we cannot exclude a small influence of the intracellular colicin concentration at this point. Interestingly, upon MitC induction we found the mean YFP fluorescence intensity values of cells expressing *cea* to be very similar and independent of the applied MitC concentration. This indicates, that as soon as an external stressor is sensed a fast response is favored, rather than the production of even higher colicin concentrations. In contrast, the average YFP fluorescence intensity (representing the colicin amount produced) per cell stays constant (S1 Table), and the colicin is released at earlier time-points at high exogenous stress levels. The intensity of the population wide response is then increased by a higher fraction of cells producing and releasing the colicin. In summary, we conclude that the level and kind of heterogeneity, in expression of the Colicin E2 operon, is adaptable to the environmental situation.

## Supporting Information

S1 Fig Definition of a threshold for the “ON” state. a) Time course of a single cell for definition of the threshold showing a cell in the ‘OFF’ state. The FI does not reach a value higher than five times the mean (indicated by the red dotted line) of the first three FI values (red straight line) and is therefore defined as ‘OFF’. b) Time course of a single cell for definition of the threshold showing a cell in the ‘ON’ state. The FI of this cell reaches at time-point t (vertical red dotted line) a value equal to five times the mean of the first three FI values (horizontal red dotted line). From that time-point t on, the cell is counted as a cell in the ‘ON’ state.
(EPS)

S2 Fig Single cell time traces of *cel* expressing cells. **A—F** Fluorescence intensity (FI) over time for single cells expressing CFP (depicts *cel* expression, grey lines) as well as the mean fluorescence intensity (black circles) of all cells at each single time-point is shown, the errorbars denote the standard error of the mean. **A:** no MitC added, **B:** 0.05 μg/ml MitC, **C:** 0.1 μg/ml MitC, **D:** 0.25μg/ml MitC, **E:** 0.4 μg/ml MitC, **F:** 0.7 μg/ml MitC.
(EPS)

S3 Fig Determination of the switching window of *cel* expressing cells in dependence of different MitC concentrations. **A—D:** Histograms of the number of cells switching into the ‘ON’ state at every time-point. The histograms are given for different MitC concentrations (**A:** 0.0 μg/ml, **B:** 0.05 μg/ml, **C:** 0.25 μg/ml, **D:** 0.7 μg/ml) The black lines denote Gaussian fits to these histograms (S6 Table). Please note, due to the small number of cells in the ‘ON’ state a Gaussian fit could not be applied for 0.0 μg/ml MitC. **E:** Time-point of maximal switching into the ‘ON’ state (x_0,_ black circles) of *cel* expression for different MitC concentrations, fitted by an exponential decay (black line, S7 Table). Switching window of *cel* expression (w, grey squares) at different MitC concentrations, fitted by an exponential function (grey line, S7 Table). The errorbars show the errors of the fits of the corresponding histograms.
(EPS)

S4 Fig Comparison of CFP (*cel*) expression in EMO3-C and EMO3-S. **A-F** Mean fluorescence intensities for CFP (*cel*) expression over time for different MitC concentrations (**A:** 0.0 μg/ml, **B:** 0.05 μg/ml, **C:** 0.1 μg/ml, **D:** 0.25 μg/ml, **E:** 0.4 μg/ml and **F:** 0.7 μg/ml). EMO3-C harboring two plasmids (pMO3 and colicinE2-P9) is depicted in black circles; EMO3-S (with pMO3 only) is displayed in grey squares. The errorbars denote the error of the mean.
(EPS)

S1 Text Determination of the maturation times of the two fluorescent proteins .
(DOCX)

S1 Table Overview of important data for *cea* expression of EMO3-C at different MitC concentrations. %ON_75min_ = cells in the ‘ON’ state at time-point t = 75min; %ON_max_ = maximal (cumulative) percentage of cells in the ‘ON’ state; Mean FI_MaxON_ = mean maximal FI of all cells in the ‘ON’ state.
(DOCX)

S2 Table Overview of important data for *cel* expression of EMO3-C at different MitC concentrations. %ON_75min_ = cells in the ‘ON’ state at time-point t = 75min; %ON_max_ = maximal (cumulative) percentage of cells in the ‘ON’ state; Mean FI_MaxON_ = mean maximal FI of all cells in the ‘ON’ state.
(DOCX)

S3 TableFit parameter for the sigmoid fits given in Fig. 2B.These data were fitted by $y\;=\;y_{0}+\left\{max/\left(1+exp\left(\frac{x_{half}-x}{r}\right)\right)\right\}$ with the y offset *y*_*0*_, the maximum *max*, the time-point of *(y*_*0*_ *+max)/2 = x*_*half*_ and the rate *r*.(DOCX)

S4 TableFit parameter for the Gaussian fits of the histograms given in Fig. 4 B–D.The data were fitted by $y=y_{0}+A*exp\left\{-\left(\frac{x-x_{0}}{w}\right)²\right\}$ with the Amplitude *A*, the y offset *y*_*0*_, the x offset *x*_*0*_ and the full width at half maximum (FWHM) *w*.(DOCX)

S5 Table Fit parameter for the single exponential fits given in Fig. 4F. These data were fitted by *y* = *y*_0_ + A*e^−τ*x^ with the Amplitude *A*, the y offset *y*_*0*_ and the rate τ.
(DOCX)

S6 TableFit parameter for the Gaussian fits of the histograms given in S3 Fig. B–D.The data were fitted by $\begin{array}{l} y=y_{0}+A*exp\left\{-\left(\frac{x-x_{0}}{w}\right)²\right\} \end{array}$ with the Amplitude *A*, the y offset *y*_*0*_, the x offset *x*_*0*_ and the full width at half maximum (FWHM) *w*.(DOCX)

S7 Table Fit parameter for the single exponential fits given in S3 Fig. E. These data were fitted by *y* = *y*_0_ + A*e^−τ*x^ with the Amplitude *A*, the y offset *y*_*0*_ and the rate τ.
(DOCX)
