# Supplementary material for: Amount of Colicin Release in Escherichia coli Is Regulated by Lysis Gene Expression of the Colicin E2 Operon
Source: PLoS One. 2015 Mar 9;10(3):e0119124. doi: 10.1371/journal.pone.0119124 (PMC4353708; doi:10.1371/journal.pone.0119124)
Supplement: S1 Text — . (DOCX) [file pone.0119124.s005.docx]

**Supporting Information**

***Maturation time***

To evaluate the maturation times of our FPs in vivo, we used a standard protocol (1, 2) and performed single cell time-lapse microscopy. After induction with MitC (45 min or 60 min for 0.4µg/ml and 0.1µg/ml MitC, respectively), translation of mRNA was inhibited via the addition of the antibiotic Chloramphenicol at a concentration of 200µg/ml. After addition of Chloramphenicol a picture was taken every 3 minutes for the next 30 minutes. An increase of fluorescence intensity can thereby only be due to maturation of already translated proteins (folding of the chromophore of the FP). The maturation times of YFP and CFP were than obtained by fitting the increase in FI using the program Igor Pro 6.2 (Wavematrics) with the function e^(-inv Tau *x) (2). To exclude the possibility that different MitC concentrations have an effect on maturation times, we performed maturation time determination measurements for low and high MitC concentrations, 0.1 and 0.4 µg/ml, respectively. As expected no significant difference could be found and the determined values are within the errors in agreement with literature values (3, 4).

References:

1. Megerle JA, Fritz G, Gerland U, Jung K, Rädler JO. Timing and Dynamics of Single Cell Gene Expression in the Arabinose Utilization System. Biophysical journal. 2008;95(4):2103-15.

2. Hebisch E, Knebel J, Landsberg J, Frey E, Leisner M. High Variation of Fluorescence Protein Maturation Times in Closely Related *Escherichia coli* Strains. PLoS ONE. 2013;8(10):e75991.

3. Kremers G-J, Goedhart J, van Munster EB, Gadella TWJ. Cyan and Yellow Super Fluorescent Proteins with Improved Brightness, Protein Folding, and FRET Förster Radius†,‡. Biochemistry. 2006;45(21):6570-80.

4. Nagai T, Ibata K, Park ES, Kubota M, Mikoshiba K, Miyawaki A. A variant of yellow fluorescent protein with fast and efficient maturation for cell-biological applications. Nat Biotech. 2002;20(1):87-90.
