# Supplementary material for: Amount of Colicin Release in Escherichia coli Is Regulated by Lysis Gene Expression of the Colicin E2 Operon
Source: PLoS One. 2015 Mar 9;10(3):e0119124. doi: 10.1371/journal.pone.0119124 (PMC4353708; doi:10.1371/journal.pone.0119124)
Supplement: S1 Table — %ON75min = cells in the ‘ON’ state at time-point t = 75min; %ONmax = maximal (cumulative) percentage of cells in the ‘ON’ state; Mean FIMaxON = mean maximal FI of all cells in the ‘ON’ state. (DOCX) [file pone.0119124.s006.docx]

| **MitC**  **[µg/ml]** | **%ON_75min_** | **%ON_max_** | **Mean FI_MaxON_**  **[FU]** |
| --- | --- | --- | --- |
| 0.0 | 2.0 ± 1.4 | 28.0 ± 4.5 | 2905 ± 1805 |
| 0.05 | 20.0 ± 4.0 | 86.0 ± 3.5 | 5089 ± 1448 |
| 0.1 | 23.0 ± 4.2 | 93.0 ± 2.6 | 5186 ± 1604 |
| 0.25 | 75.0 ± 4.3 | 99.0 ± 1.0 | 5138 ± 1454 |
| 0.4 | 74.0 ± 4.4 | 93.0 ± 2.6 | 4794 ± 1543 |
| 0.7 | 64.0 ± 4.8 | 90.0 ± 3.0 | 4592 ± 1590 |
