# Supplementary material for: Amount of Colicin Release in Escherichia coli Is Regulated by Lysis Gene Expression of the Colicin E2 Operon
Source: PLoS One. 2015 Mar 9;10(3):e0119124. doi: 10.1371/journal.pone.0119124 (PMC4353708; doi:10.1371/journal.pone.0119124)
Supplement: S2 Table — %ON75min = cells in the ‘ON’ state at time-point t = 75min; %ONmax = maximal (cumulative) percentage of cells in the ‘ON’ state; Mean FIMaxON = mean maximal FI of all cells in the ‘ON’ state. (DOCX) [file pone.0119124.s007.docx]

| **MitC**  **[µg/ml]** | **%ON_75min_** | **%ON_max_** | **Mean FI_MaxON_**  **[FU]** |
| --- | --- | --- | --- |
| 0.0 | 1.0 ± 1.0 | 26.0 ± 4.4 | 2533 ± 1474 |
| 0.05 | 19.0 ± 3.9 | 95.0 ± 2.2 | 3455 ± 1490 |
| 0.1 | 15.0 ± 3.6 | 95.0 ± 2.2 | 3444 ± 1575 |
| 0.25 | 64.0 ± 4.8 | 100.0 ± 0.0 | 3135 ± 1275 |
| 0.4 | 68.0 ± 4.7 | 98.0 ± 1.4 | 3173 ± 1485 |
| 0.7 | 65.0 ± 4.8 | 92.0 ± 2.7 | 2933 ± 1258 |
