# Supplementary material for: Amount of Colicin Release in Escherichia coli Is Regulated by Lysis Gene Expression of the Colicin E2 Operon
Source: PLoS One. 2015 Mar 9;10(3):e0119124. doi: 10.1371/journal.pone.0119124 (PMC4353708; doi:10.1371/journal.pone.0119124)
Supplement: S3 Table — These data were fitted by y = y0+{max/(1+exp(xhalf−xr))} with the y offset y 0, the maximum max, the time-point of (y 0 +max)/2 = x half and the rate r. (DOCX) [file pone.0119124.s008.docx]

|  | **X_half_ [µg/ml]** | **r [1/(µg/ml)]** | **max [%]** | **y_0_ [%]** |
| --- | --- | --- | --- | --- |
| *cea* | 0.13 ± 0.04 | 0.04 ± 0.05 | 65.89 ± 17.6 | 5.6 ± 14.2 |
| *cel* | 0.15 ± 0.04 | 0.04 ± 0.03 | 61.3 ± 12.0 | 5.8 ± 9.4 |
