# Supplementary material for: Amount of Colicin Release in Escherichia coli Is Regulated by Lysis Gene Expression of the Colicin E2 Operon
Source: PLoS One. 2015 Mar 9;10(3):e0119124. doi: 10.1371/journal.pone.0119124 (PMC4353708; doi:10.1371/journal.pone.0119124)
Supplement: S4 Table — The data were fitted by y=y0+A*exp{−(x−x0w)²} with the Amplitude A, the y offset y 0, the x offset x 0 and the full width at half maximum (FWHM) w. (DOCX) [file pone.0119124.s009.docx]

| **MitC [µg/ml]** | **x_0_ [min]** | **w [min]** | **A [#]** | **y_0_ [#]** |
| --- | --- | --- | --- | --- |
| 0.05 | 101.2 ± 4.32 | 51.52 ± 7.63 | 12.70 ± 1.43 | 0.44 ± 0.81 |
| 0.25 | 69.71 ± 0.77 | 18.97 ± 1.17 | 43.80 ± 2.69 | 0.08 ± 0.67 |
| 0.7 | 60.44 ± 0.41 | 11.18 ± 0.32 | 57.62 ± 1.37 | 0.46 ± 0.32 |
