# Supplementary material for: Amount of Colicin Release in Escherichia coli Is Regulated by Lysis Gene Expression of the Colicin E2 Operon
Source: PLoS One. 2015 Mar 9;10(3):e0119124. doi: 10.1371/journal.pone.0119124 (PMC4353708; doi:10.1371/journal.pone.0119124)
Supplement: S5 Table — These data were fitted by y = y 0 + A*e−τ*x with the Amplitude A, the y offset y 0 and the rate τ. (DOCX) [file pone.0119124.s010.docx]

| **Item** | τ **[ml/µg]** | **A [min]** | **y_0_ [min]** |
| --- | --- | --- | --- |
| time-point maximal switching | 6.45 ± 1.43 | 56.90 ± 4.8 | 60.57 ± 2.66 |
| time window of switching | 19.3 ± 6.1 | 95.5 ± 31.4 | 14.9 ± 2.23 |
