# Supplementary material for: Amount of Colicin Release in Escherichia coli Is Regulated by Lysis Gene Expression of the Colicin E2 Operon
Source: PLoS One. 2015 Mar 9;10(3):e0119124. doi: 10.1371/journal.pone.0119124 (PMC4353708; doi:10.1371/journal.pone.0119124)
Supplement: S6 Table — The data were fitted by y=y0+A*exp{−(x−x0w)²} with the Amplitude A, the y offset y 0, the x offset x 0 and the full width at half maximum (FWHM) w. (DOCX) [file pone.0119124.s011.docx]

| **MitC [µg/ml]** | **x_0_ [min]** | **w [min]** | **A [#]** | **y_0_ [#]** |
| --- | --- | --- | --- | --- |
| 0.05 | 109.8 ± 5.79 | 58.64 ± 11.0 | 11.23 ± 1.55 | 0.77 ± 1.02 |
| 0.25 | 74.21 ± 0.68 | 20.58 ± 0.92 | 37.74 ± 1.43 | 0.41 ± 0.39 |
| 0.7 | 65.07 ± 0.54 | 11.47 ± 1.13 | 61.16 ± 4.96 | 0.46 ± 0.46 |
