# Supplementary material for: Amount of Colicin Release in Escherichia coli Is Regulated by Lysis Gene Expression of the Colicin E2 Operon
Source: PLoS One. 2015 Mar 9;10(3):e0119124. doi: 10.1371/journal.pone.0119124 (PMC4353708; doi:10.1371/journal.pone.0119124)
Supplement: S7 Table — These data were fitted by y = y 0 + A*e−τ*x with the Amplitude A, the y offset y 0 and the rate τ. (DOCX) [file pone.0119124.s012.docx]

| **Item** | τ **[ml/µg]** | **A [min]** | **y_0_ [min]** |
| --- | --- | --- | --- |
| time-point maximal switching | 6.52 ± 1.42 | 64.31 ± 5.36 | 64.50 ± 2.92 |
| time window of switching | 25.43 ± 8.7 | 150.3 ± 67.9 | 16.43 ± 2.66 |
